# Complete Genomic and Ultrastructural Analysis of a Nam Dinh Virus Isolated from *Culex pipiens quinquefasciatus* in China*

**DOI:** 10.1038/s41598-017-00340-3

**Published:** 2017-03-21

**Authors:** Jianming Zhou, Yujuan Jin, Yingjian Chen, Jingmei Li, Qiwen Zhang, Xianqing Xie, Liping Gan, Qu Liu

**Affiliations:** Longgang Center for Disease Control and Prevention in Shenzhen, Shenzhen, China

## Abstract

The Nam Dinh virus (NDiV) was isolated from *Culex quinquefasciatus* in Shenzhen, China, for the first time, in 2011. In this study, we characterized the ultrastructure of NDiV, determined its complete genome sequence and made comparisons with other known nidoviruses. Electron microscopic observation revealed that the NDiV strain isolated in China produced viral nucleocapsid-like particles and vesicles in host cells. The extracellular virions were enveloped and were spherical with short spikes. The complete genome sequence of the newly isolated NDiV was submitted to the GenBank database (GenBank accession number KF522691). Sequencing of the viral genome showed that the homologies of NDiV isolated in China and Vietnam were greater than 94.0% and 89.0% at the nucleotide and amino acid sequence levels, respectively. Moreover, gene substitution was detected, whereas insertions and deletions were not. A phylogenetic tree analysis showed that these viruses belong to the genus *Alphamesonivirus1* of the family *Mesoniviridae*. The similarity between the two viruses regarding morphological and molecular biological characteristics indicates that the molecular genetics of NDiV are conservative and that the regional differences are unlikely to have a significant effect. This is the first report of the isolation and complete sequencing of a mesonivirus in mainland China.

## Introduction

A virus thought to be the first known insect-borne nidovirus was discovered during surveillance for Japanese encephalitis virus (JEV) in mosquitoes from Vietnam^1^. The Nam Dinh virus (NDiV) was identified in mosquitoes, and no evidence of vertebrate infection by this virus has been detected. NDiV was found to be a positive-sense, single-stranded RNA ([+]ssRNA) virus. The 20,192 nucleotide (nt)-long genome contains two major open reading frames (ORFs), ORF1a and ORF1b, which encode two proteins (pp1a and pp1ab, respectively) using a ribosomal frame shift mechanism. The intact NDiV virion exhibited a spherical form with a diameter of 60–80 nm^2^. The structure, replication and transcription patterns of the viral genome were similar to those of coronaviruses in the order *Nidovirales*. A phylogenetic analysis showed that NDiV is distantly related to coronaviruses, indicating that this virus represents a missing link in the transition from small to large nidoviruses.

A newly established family, *Mesoniviridae*, which is based on two closely related viruses, the Cavally virus (CavV) and NDiV, has been classified as a new member of *Nidovirales* by the Executive Committee of the International Committee on Taxonomy of Viruses (ICTV)^3^. At present, ten mesoniviruses have been successfully isolated, including CavV, the Hana virus (HanaV), the Meno virus (MenoV), the Nse virus (NseV) and the Moumou virus (MoumouV) in Cote d’Ivoire, NDiV in Vietnam and America, the Dak Nong virus (DKNV) in Vietnam, the Bontang Baru virus (BBaV) and the Karang Sari virus (KSaV) in Indonesia, the Kamphaeng Phet virus (KPhV) in Thailand and the Casuarina virus (CASV) in Australia^1, 4–8^. Mesoniviruses are widely distributed in different geographic regions and have a large host range of mosquito species. Thus, these viruses are commonly detected in mosquitoes around the world, with mesoniviruses likely to be present in more area than other viruses^4^.

NDiV has been successfully isolated from various mosquito species in Vietnam and the United States. Further investigations of the various features of the ecology, morphology and molecular biology of this virus are critical for an improved understanding of the virus. Furthermore, studies of NDiV as part of mosquito monitoring programs in a greater number of areas are required to enrich the resources available for such research.

During the period from 2009 to 2012, NDiV was isolated from samples of *Culex pipiens quinquefasciatus* in Shenzhen China^9^. In this study, we report on the successful isolation of the ultrastructure of NDiV for the first time in China and its complete genome sequence. We also present comparisons of the nt and amino acid (aa) sequences of NDiV with those of other viruses and a phylogenetic analysis.

## Results

### Distribution of mosquitoes

During arbovirus surveillance of mosquitoes in the Longgang District of Shenzhen, China, a total of 18,560 mosquitoes belonging to seven species (*Aedes albopictus*, *Aedes togoi*, *C. pipiens quinquefasciatus*, *Culex tritaeniorhynchus*, *Anopheles sinensis*, *Anopheles minimus* and *Anopheles anthropophagus*) and representing three genera (*Aedes*, *Culex* and *Anophelinae*) were collected. Of these, 95.2% were *C. pipiens quinquefasciatus* (17,404; 93.8%) or *Culex tritaeniorhynchus* (278; 1.5%).

### Virus isolation and identification by PCR

A total of 220 pools containing all the collected mosquitoes and 4 pools of *C. pipiens quinquefasciatus* collected from hospitals (SZ10618Z/2010.6, SZ11706Z/2011.7) and residential dwellings (SZ11714Z/2011.7, SZ11826Z/2011.8) caused cytopathic effects (CPEs) in C6/36 cells and were positive for the putative RNA-dependent RNA polymerase (RdRp) gene of NDiV; however, they did not cause CPEs in baby hamster kidney (BHK-21) cells. The nt and aa distances of the RdRp gene of the isolate SZ11706Z and three other isolates were 0.11 ± 0.06% and 0.20 ± 0.08%, respectively. This suggested that the four isolates should be classified as a single virus. Further analysis of one of these isolates (SZ11706Z) is described below.

### Electron microscopy

**Figure 1:**
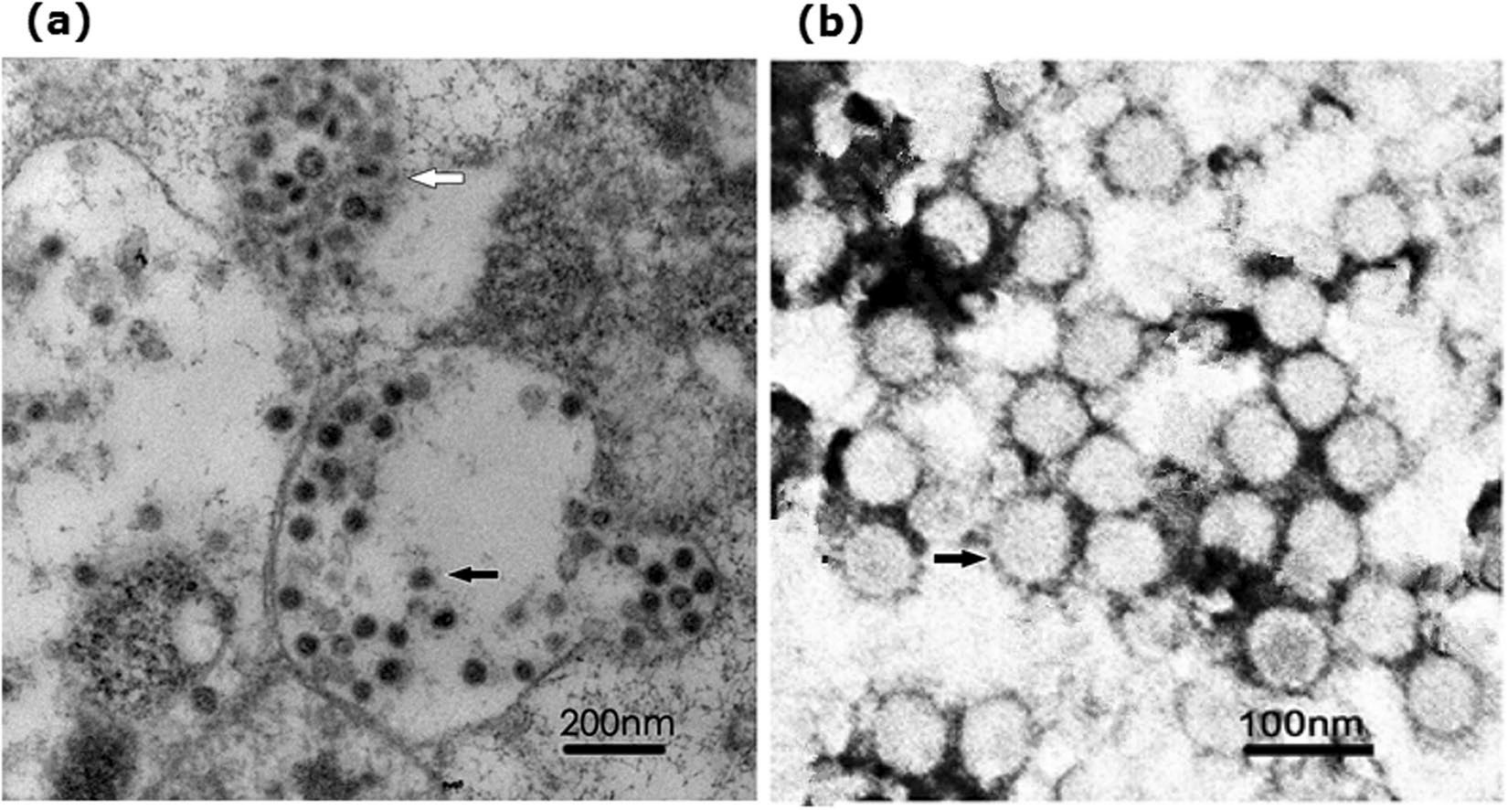
Ultrastructural characteristics of NDiV-SZ11706Z. (**a**) Ultrathin sections of NDiV in a cytoplasmic vacuole (black arrow) and a higher magnification of vesicles containing numerous virions (white arrow). (**b**) Electron micrograph of negatively stained NDiV virions.

To observe the morphological characteristics of the SZ11706Z strain, we used ultrathin sections and negative staining for the electron microscopic examination. Viral nucleocapsid-like particles and numerous vesicle-enveloped particles (40–60 nm in diameter without surface projections) were detected in the cytoplasm of infected insect cells at 24 h post-infection (Fig. 1a). Observation of negatively stained samples by electron microscopy revealed the presence of enveloped, spherical virions with typical NDiV morphology in the culture supernatants of infected cells at 48 h post-infection. These virions ranged from 50 to 80 nm in diameter and displayed very short spikes approximately 3 nm in length (Fig. 1b).

### Complete viral genome organization

The complete genome sequence of the SZ11706Z strain comprised 20,130 nt, including a 5′-untranslated region (UTR) (1–360 nt) followed by at least six ORFs: ORF1a (361–7,869 nt), ORF1b (7,830–15,635 nt), ORF2a (15,660–18,356 nt), ORF2b (15,674–16,309 nt), ORF3 (18,449–18,877 nt) and ORF4 (18,756–19,103 nt) and a 3′-UTR (19,104–20,130 nt), excluding the 3′ poly (A) tail. The “slippery sequence” GGAUUUU that is present in NDiV and controls ORF1a/ORF1b −1 ribosomal frame shifting (RFS) was also conserved in the SZ11706Z strain.

### Phylogenetic and sequence analysis

Analyses of the nt and deduced aa homologies of the six conserved protein domains and ORFs of SZ11706Z and other representative mesoniviruses were performed using BLAST searches. Sequence alignment showed that the SZ11706Z strain shared the greatest degrees of nt and aa similarity with NDiV/02VN178/Vietnam (94–99.0% and 89–99.0%, respectively). The superfamily 1 helicase (Hel1) domain that is conserved in all nidoviruses had a higher aa identity than other protein domains, ranging from 79.0 to 99.0% (Table 1). Compared to the ORFs of NDiV-02VN178, ORF3b of the SZ11706Z strain, which contained 13 site-specific mutations, displayed the lowest degree of aa sequence conservation (89.0%) (Table 2).

**Table 1:** Comparison of six critical replicase domains and the complete ORFs of NDiV-SZ11706Z with those of other mesoniviruses.

	3CL^pro^	RdRp	Hel1	ExoN	NMT	OMT	ORF1ab	ORF1a	ORF2a	ORF2b	ORF3a	ORF3b
NDiV-SZ11706Z	804* (268)	1671 (557)	1212 (404)	807 (269)	699 (233)	723 (241)	15276 (5092)	7512 (2503)	2697 (899)	636 (212)	429 (143)	348 (116)
NDiV-02VN178	99.0^#^ (99.0)	99.0 (99.0)	99.0 (99.0)	99.0 (99.0)	99.0 (99.0)	99.0 (98.0)	99.0 (99.0)	99.0 (99.0)	99.0 (98.0)	99.0 (99.0)	96.0 (97.0)	94.0 (89.0)
DKNV	83.0 (90.0)	87.0 (92.0)	86.0 (93.0)	83.0 (84.0)	83.0 (83.0)	86.0 (86.0)	81.0 (88.0)	76.0 (72.0)	90.0 (86.0)	88.0 (76.0)	94.0 (95.0)	94.0 (91.0)
CavV	90.0 (97.0)	93.0 (98.0)	92.0 (98.0)	93.0 (96.0)	92.0 (94.0)	91.0 (94.0)	91.0 (93.0)	88.0 (90.0)	91.0 (88.0)	90.0 (89.0)	90.0 (92.0)	92.0 (83.0)
HanaV	83.0 (89.0)	84.0 (87.0)	86.0 (88.0)	86.0 (87.0)	87.0 (90.0)	81.0 (76.0)	82.0 (84.0)	80.0 (82.0)	87.0 (82.0)	84.0 (79.0)	89.0 (87.0)	87.0 (74.0)
MenoV	68.0 (74.0)	74.0 (76.0)	78.0 (80.0)	70.0 (64.0)	80.0 (69.0)	73.0 (68.0)	72.0 (60.0)	70.0 (50.0)	70.0 (61.0)	72.0 (51.0)	72.0 (63.0)	79.0 (50.0)
NseV	74.0 (79.0)	76.0 (80.0)	74.0 (79.0)	75.0 (70.0)	76.0 (69.0)	77.0 (70.0)	73.0 (67.0)	71.0 (60.0)	77.0 (67.0)	78.0 (60.0)	79.0 (84.0)	72.0 (60.0)
MoumoV	—	78.0 (83.0)	76.0 (80.0)	74.0 (68.0)	74.0 (70.0)	75.0 (70.0)	—	—	—	—	—	—

*Nucleotide number (amino acid number); ^#^percent nucleotide sequence homology (amino acid sequence homology).

**Table 2:** Deduced amino acid substitutions in ORF3b of NDiV-SZ11706Z and NDiV-02VN178.

Strain	ORF3b												
	aa-7	aa-15	aa-16	aa-17	aa-19	aa-26	aa-38	aa-39	aa-40	aa-41	aa45	aa-48	aa-50
NdiV-SZ11706Z	C (Cys)	V (Val)	K (Lys)	T (Thr)	I (Ile)	D (Asp)	A (Ala)	D (Asp)	T (Thr)	S (Ser)	A (Ala)	N (Asn)	D (Asp)
NdiV-02VN178	F (Phe)	Q (Gln)	N (Asn)	N (Asn)	T (Thr)	G (Gly)	D (Asp)	R (Arg)	I (Ile)	N (Asn)	S (Ser)	Q (Gln)	E (Lys)

**Figure 2:**
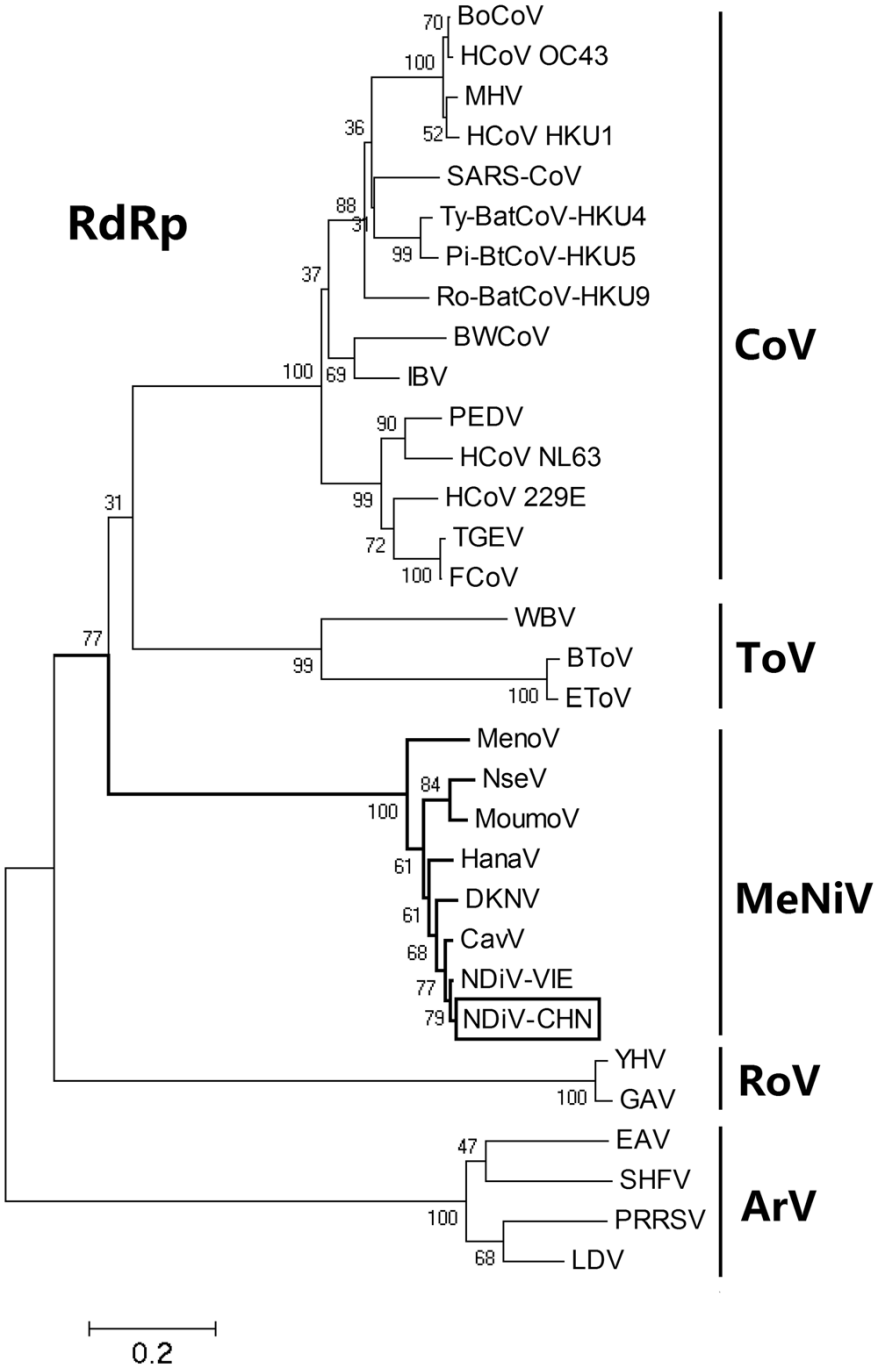
Phylogenetic analysis of nidoviruses based on the deduced amino acid sequences of RdRp domains. The phylogenetic tree was produced by the neighbor-joining (NJ) method and constructed with 1,000 bootstrap replications. The scale indicates the number of amino acid substitutions. The tree was rooted using the arterivirus branch. The GenBank/Refseq accession numbers of the nidovirus species are as follows: BoCoV, bovine coronavirus (NC_003045); HCoV OC43, human coronavirus OC43 strain ATCC VR-759 (AY585228); MHV, murine hepatitis virus strain A59 (AY700211); HCoV HKU1, human coronavirus HKU1 genotype B (AY884001); SARS-CoV, SARS coronavirus CUHK-AG03 (AY345988); Ty-BatCoV-HKU4, bat coronavirus HKU4-1 (EF065505); Pi-BtCoV-HKU5, bat coronavirus HKU5-1 (EF065509); Ro-BatCoV-HKU9, bat coronavirus HKU9-1 (EF065513); BWCoV, Beluga whale coronavirus SW1 (NC_010646); IBV, avian infectious bronchitis virus (NC_001451); PEDV, porcine epidemic diarrhea virus (NC_003436); HCoV NL63, human coronavirus NL63 isolate Amsterdam 057 (DQ445911); HCoV 229E, human coronavirus 229E (NC_002645); TGEV, TGEV Purdue P115 (DQ811788); FCoV, feline coronavirus (NC_007025); WBV, white bream virus (NC_008516); BToV, Breda virus (NC_007447); EToV, Berne virus (X52374); MenoV, Meno virus strain E9/CI/2004 (JQ957873); NseV, Nse virus strain F24/CI/2004 (JQ957874); MoumouV, Moumou virus strain C88/CI/2004 (KC768950); HanaV, Hana virus strain A4/CI/2004 (JQ957872); DKNV, Dak Nong virus (AB753015); CavV, Cavally virus isolate C79 (HM746600); NDiV-VIE, Nam Dinh virus isolate 02VN178 (DQ458789); NDiV-CHN, Nam Dinh virus isolate SZ11706Z (KF522691); YHV, yellow head virus (EU487200); GAV, gill-associated virus (NC_010306); EAV, equine arteritis virus (NC_002532); SHFV, simian hemorrhagic fever virus (NC_003092); PRRSV, porcine reproductive and respiratory syndrome virus isolate PA8 (AF176348); LDV, lactate dehydrogenase-elevating virus Plagemann strain (U15146).

**Figure 3:**
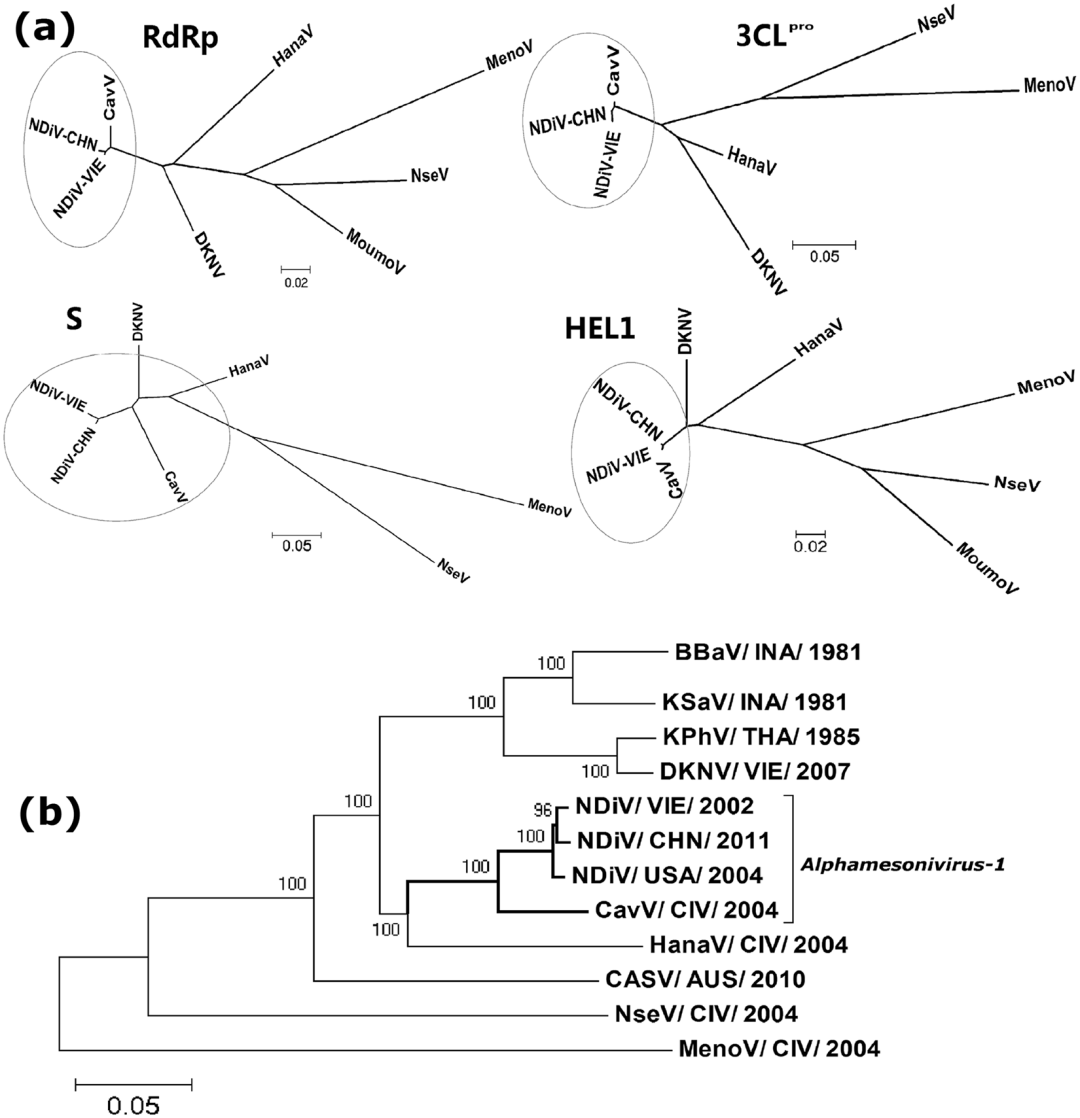
Neighbor-joining (NJ) phylogenetic trees of the members of the family Mesoniviridae. (**a**) Phylogenetic comparison of mesoniviruses based on the amino acid sequences of the conserved domains of proteins including 3C-like chymotrypsin-like protease (3CL^pro^), superfamily 1 helicase (Hel1), putative RNA-dependent RNA polymerase (RdRp) and putative spike glycoprotein (S). (**b**) Evolutionary relationships of the putative proteins 1ab of 9 putative mesonivirus species (*Alphamesonivirus*-*1*-*9*). The virus names and GenBank accession numbers are as follows: NDiV/CHN/2011, Nam Dinh virus isolate SZ11706Z (KF522691); NDiV/VIE/200, Nam Dinh virus isolate 02VN178 (DQ458789); NDiV/USA/2004, Nam Dinh virus isolate V3872 (KC807175); CavV/CIV/2004, Cavally virus isolate C79 (HM746600); MenoV/CIV/2004, Meno virus strain E9/CI/2004 (JQ957973); NseV/CIV/2004, Nse virus strain F24/CI/2004 (JQ957874); HanaV/CIV/2004, Hana virus strain A4/CI/2004 (JQ957872); DKNV/VIE/2007, Dak Nong virus (AB753015); CASV/AUS/2010, Casuarina virus (KJ125489).

Using the neighbor-joining (NJ) method, a phylogenetic tree of nidoviruses was constructed based on the conserved protein domains of RdRp. As shown in Fig. 2, the deduced aa sequence of the NDiV-CHN (SZ11706Z) RdRp gene aligned well with the major nidovirus RdRp sequence, suggesting that the newly isolated NDiV-CHN is closely related to NDiV-VIE (02VN178) isolated from *Culex triaeniorhynchus* in Vietnam and that it belongs to the family *Mesoniviridae*. A phylogenetic analysis of the viral strains of mesoniviruses around the world showed that NDiV-CHN was grouped into the type species of *Alphamesonivirus 1*, which was formed by NDiV and CavV (Fig. 3a and b).

## Discussion

NDiV has been successfully isolated from various mosquito species including *Culex vishnui* from Vietnam^1^, *C. triaeniorhynchus* from Vietnam, *C. pipiens quinquefasciatus* from Shenzhen, China and Houston, Texas, USA, and *A. albopictus* from Houston, Texas, USA^4^. Thus, it is most frequently detected in *Culex* mosquitoes, particularly *C. pipiens quinquefasciatus*, indicating that *Culex* mosquitoes are probably the major host of NDiV in nature. An analysis of 39 CavV isolates from different habitat types along an anthropogenic disturbance gradient showed an increase in virus prevalence from natural to modified habitat types^10^. The data for CavV isolates support the hypothesis that disturbed habitats, particularly human settlements, support the proliferation of endemic viruses more efficiently than undisturbed primary forest. As in our study, NDiV-positive adult mosquitoes were also collected in or near areas of human habitation in the USA, Indonesia and Vietnam. The increasing trend of NDiV movement from natural to modified habitat types, similar to CavV of the same species, *Alphamesonivirus 1*, requires further confirmation based on statistical evidence obtained by expanding the types of sites sampled and increasing the number of mosquito samples.

Kuwata^5^ reported that the replication and growth of DKNV found in Vietnam was supported by the cell lines C6/36, NIAS-AeAI-2 and NIID-CTR, whereas replication was not supported by the vertebrate cell lines Vero, BHK-21, HmLu-1 and CCL-141. Replication of CavV, HanaV, MenoV and NseV found in Cote d’Ivoire was detected in infected C6/36 cells but not in the other vertebrate cell lines tested^6^. Growth of the NDiV-02VN178 and SZ11706Z strains was detected only in C6/36 cells^1^, indicating a greater susceptibility of insects than of vertebrates to infection by mesoniviruses. During the period from 2005 to 2001, Nga isolated NDiV from the cerebrospinal fluid of acute encephalitis syndrome patients without JEV infection in the C6/36 cell line^11^, indicating that NDiV infects vertebrates. Thus, it is necessary to conduct further investigations of the molecular epidemiology of NDiV in patients with acute encephalitis syndrome (AES) of unknown etiology.

Nga identified NDiV as a single-stranded RNA virus based on an analysis of the ultrastructural characteristics of the virus as viewed in ultrathin sections. Furthermore, the replication of NDiV was found to occur mainly in the cytoplasm of host cells^2^. Our results also showed that the replication of NDiV isolated in China occurred in the cytoplasm of host cells rather than in cytoplasts. The vacuoles of infected cells in our study appeared similar to those described by Nga^2^, with the accumulation of viral nucleocapsid-like particles and individual spherical nucleocapsid-like particles with electron-dense interiors containing the viral genetic material observed in the cytoplasm. This indicates that the morphology of nucleocapsid-like particles of NDiV in C6/36 cell cytoplasm from the two areas is identical. *Alphamesonivirus 1*, as the type species of *Alphamesonvirus* (the new genus of *Mesoniviridae*), was proposed to recognize NDiV and CavV. The morphology of NDiV virions from China and Vietnam appeared similar in electron microscopy after negative staining. These virions were of homogeneous spherical shape and appeared to contain very short, spike-like projections. The virions of CavV were typical CoV-like virions with rod-shaped surface projections. However, there were significant differences in the appearance of the spike proteins of the two viruses, and further investigations are required to understand the relationship between the efficiency of viral envelope assembly and spike proteins.

Comparison of the aa sequences of the six major ORFs of NDiV-SZ1106Z and NDiV-02VN178 revealed differences that were due predominantly to substitution mutation; neither insertion nor deletion mutations were present. The lowest homology was identified in ORF3b.

Although Zirkel *et al*. speculated that ORF3b is involved in the synthesis of mRNA3 and in encoding membrane (M) and envelope (E) proteins, its specific role has not yet been determined^7^. Further investigations are necessary to elucidate the effects on protein synthesis and structure of the aa sequence differences encoded by the ORF3b of the viruses isolated in the two areas.

The typing of coronaviridae species is usually based on aa sequence homology with the 3C-like chymotrypsin-like protease (3CL^pro^), RdRp and Hel1 domains, which are conserved in all nidoviruses^12^. In this study, we found that the homology of NDiV-SZ11706Z and NDiV-02VN178 with CavV was greater than 90.0% based on the aa sequences of 3CL^pro^, RdRp and Hel1. The phylogenetic positions of the viruses were compared based on the 3CL^pro^, RdRp and Hel1 domains that are conserved in mesoniviruses and on the sequences of putative proteins 1ab of other representative species of mesoniviruses. Our analysis showed that NDiV-SZ11706Z and NDiV-02VN178 share a common ancestor node and form a separate rooted branch. These results support the classification of NDiV-SZ11706Z as a species of *Alphamesonivirus 1*.

At present, the genus *Alphamesonivirus* of *Mesoniviridae* includes 9 species according to the species demarcation criteria previously established for mesoniviruses. Interestingly, in the area where the first species of *Alphamesonivirus* was found, a new species of *Alphamesonivirus* was later found. For example, after CavV (*Alphamesonivirus-1*) was found in Cote d’Ivoire, new species of *Alphamesonivirus* including HanaV, MenoV and NseV (*Alphamesonivirus-2*,*3*,*4*) were found in succession^6^. After NDiV was found in the province of Nam Dinh in northern Vietnam, DKNV (Alphamesonivirus-8) was identified in the province of Dak Nong in southern Vietnam^5^. In this study, we successfully isolated NDiV from a location in Shenzhen, China. This suggests that the new species of *Alphamesonivirus* may exist in southern China.

## Conclusion

This study demonstrates that NDiV can be isolated from mosquitoes in China and provides further evidence that the virus is prevalent in mosquito populations around the world. NDiV isolated in China shows a major difference in the ORF3b region compared to NDiV isolated in Vietnam, although no morphological differences between the two isolates were apparent based on observation by electron microscopy. The similarity between the two viruses in morphological and molecular biological characteristics indicates that the molecular genetics of NDiV are conserved and that regional differences are unlikely to have a significant impact. However, further studies involving animal experiments are required to investigate potential differences in the pathogenicity of NDiV.

## Methods

### Mosquito collection

Arbovirus surveillance was conducted in the Longgang District of Shenzhen, China, during the period from August 2009 to September 2011. UV light traps (12V; 300 mA; Wuhan Lucky Star Environmental Protection Tech Co., Hubei, China) were used to capture mosquitoes ten days per month. Adult mosquitoes were collected from 38 monitoring sites that represented six habitat types, including residential dwellings, hospitals, schools, construction sites, parks and stadiums. The traps were set up at 07:00 pm, and the mosquitoes were collected at 09:00 am the following morning. The mosquitoes were separated according to species, site and collection date and were then divided into pools of approximately 100 mosquitoes each. The mosquito pools were stored in liquid nitrogen prior to virus isolation.

### Cell culture and virus isolation

*A. albopictus* C6/36 cells and BHK-21 cells were used to isolate viruses from 220 pools of 18,560 mosquitoes. Specifically, suspensions of mosquito homogenates were filtered through 0.22-µm Millipore Millex-GS filters (Millipore Corp, Billerica, MA, USA) and used to infect C6/36 and BHK-21 cells at 28 °C and 37 °C, respectively, under 5% CO_2_. CPEs were evaluated every 8 h in three blind passages after incubation for 24 h and observation over the next 7 days. A positive virus isolation from a specimen was defined as one in which 70% CPE was observed at 2 days post-infection.

### PCR detection of NDiV

Suspensions of cells showing CPE were processed for RNA extraction with the High Pure Viral RNA Kit (Invitrogen, Karlsruhe, Germany) according to the manufacturer’s instructions. A reverse transcription-polymerase chain reaction (RT-PCR) method was used for NDiV-specific gene (RdRp) detection with the Prime Script RT-PCR Kit (TaKaRa Biotechnology, Dalian, China). The primer sequences were: forward primer PRd1 5′-TCACACAACCGCATGCTACGC-3′ and reverse primer PRd2 5′-GTGGTCACGGCCAGTGGTGT-3′ (targeting a 2394 bp fragment). The polymerase chain reaction (PCR) products were visualized on 2% agarose gels and extracted from the gels using the PCR Purification Combo Kit (Invitrogen). The PCR fragments were sequenced and compared to known RdRp gene sequences of NDiV in the National Center for Biotechnology Information (NCBI) GenBank database by BLASTN.

### Electron microscopic analysis

The NDiV-infected cells were collected and prepared for electron microscopy. Ultrathin sections and negatively stained material were evaluated by electron microscopy on a Tecnai 12 transmission electron microscope as described previously^2^.

### Complete viral genome sequencing

Total viral RNA was used as a template for cDNA synthesis by RT-PCR using the SuperScript III First-Strand Synthesis System (Invitrogen). PCR was performed with twenty-two pairs of overlapping primers that were designed based on the complete genome sequence of NDiV-02VN178 (GenBank accession number DQ458789) (Table S1). The PCR products were further purified using a Pure Link Quick Gel Extraction and PCR Purification Combo Kit (Invitrogen) and directly sequenced on both strands using an automated DNA sequencer (Applied Biosystems 3730xl DNA Analyzer, USA).

### Phylogenetic and sequence analysis

Pairwise analyses of the newly identified NDiV at the nt and aa levels were performed using DNASTAR software. Alignment and phylogenetic analyses were performed by comparison of the RdRp motif domains with those of other nidoviruses and by comparison of the conserved regions within 3CL^pro^, HEL1, RdRp, the putative spike glycoprotein (S) and putative proteins 1ab with those of other mesoniviruses. An NJ tree was established with 1.000 bootstrap replicates in MEGA 4.1. The flowchart for isolation and identification of NDiV is shown in Figure S1.

### Nucleotide sequence accession numbers

The full genome sequence of strain SZ11706Z was assigned the GenBank accession number KF522691. The RdRp gene sequences of strains SZ10618Z, SZ11706Z, SZ11714Z and SZ11826Z were assigned the GenBank accession numbers JQ996712 to JQ996715, respectively.

## Electronic supplementary material

Supplementary Table S1 and Figure S1.
