# Supplementary material for: Complete Genomic and Ultrastructural Analysis of a Nam Dinh Virus Isolated from Culex pipiens quinquefasciatus in China*
Source: Sci Rep. 2017 Mar 21;7:271. doi: 10.1038/s41598-017-00340-3 (PMC5428213; doi:10.1038/s41598-017-00340-3)
Supplement: Supplementary file 1 — Supplementary Table S1 and Figure S1. [file 41598_2017_340_MOESM1_ESM.pdf]

Supplementary Information

Title: Complete Genomic and Ultrastructural analysis of a Nam Dinh Virus Isolated from Culex pipiens quinquefasciatus in China

Authors: Jianming Zhou, Yujuan Jin, Yingjian Chen, Jingmei Li, Qiwen Zhang, Xianqing Xie, Liping Gan and Qu Liu.

Supplementary Table S1

Supplementary Figure S1

Table S1

Table S1. The primer sets for amplifying the complete genome of NDiV

Primer	Sequence,5'-3'	Position
NDiV-SZ-1-F	ACTAAAGAAACTTTTGTTTTCTCCCATAAT	1–30
NDiV-SZ-1-R	CATAGTGGCGTCATCTAGCTCG	1107–1128
NDiV-SZ-2-F	GCGAACTTACCACTATTGAATCTAACCT	944–971
NDiV-SZ-2-R	CATGTCTTACGACAGGAGGGG	2007–2027
NDiV-SZ-3-F	CCTGCTTGCATTCTATTCACCG	1888–1909
NDiV-SZ-3-R	TGGTCTTCCAGGAGCTGC	2931–2948
NDiV-SZ-4-F	AGTCTTTGTTTTGGGCACGC	2817–2836
NDiV-SZ-4-R	TGTGGCCAATTGCAAGATG	4068–4086
NDiV-SZ-5-F	GGACCACTGTTGCTAACACCT	3886–3906
NDiV-SZ-5-R	CAGCGCAATGCAAGCCTA	5024–5041
NDiV-SZ-6-F	TTGTCACGTTACGACACGGAA	4888–4908
NDiV-SZ-6-R	GTTAAAGGCGGCGTAGGGT	5883–5901
NDiV-SZ-7-F	GCCATCCGCTACAATCCCTA	5746–5765
NDiV-SZ-7-R	TACCTGTCCATAGTATTGTCGTGAGT	6864–6889
NDiV-SZ-8-F	TTCTCGGCTCTGACCTGGA	6752–6770
NDiV-SZ-8-R	TGTTCCTGCTTAGAGTACGAGCC	7859–7881
NDiV-SZ-9-F	AACTTACGCAGACAACACCGTTA	7716–7738
NDiV-SZ-9-R	TGCGGACAATCCATAGTATCG	8762–8782
NDiV-SZ-10-F	AATCGGCAGTTGAGGCTACTCG	8617–8638
NDiV-SZ-10-R	GGTATGGAGTACCGGCTGAT	9701–9720
NDiV-SZ-11-F	ATCAGAACACCGCTGGCTC	9550–9568
NDiV-SZ-11-R	CATAGCGTTGGATAGCGTCGAT	10656–10677
NDiV-SZ-12-F	ACAATTCGCCGACGCGTA	10506–10523
NDiV-SZ-12-R	TGTCACACGCTTTGTGTCTGTTAC	11496–11519
NDiV-SZ-13-F	TATACACCACTGGCCGTGACC	11386–11406
NDiV-SZ-13-R	GTGCAAGGCCGGTTAGTAGG	12551–12570

NDiV-SZ-14-F	CAAAGACCGCATTTGCGTGA	12443–12462
NDiV-SZ-14-R	ATAGCGCACTGGAATGCCTG	13546–13565
NDiV-SZ-15-F	CAATGAACATAGTTATGGACGACTGTA	13420–13446
NDiV-SZ-15-R	AAGCCAGTGGTCCCGAGTTT	14589–14608
NDiV-SZ-16-F	AATCCACAGAATAACATGAACACC	14454–14477
NDiV-SZ-16-R	TGTGATGTCCAGCCTTGTACG	15512–15532
NDiV-SZ-17-F	GGAGCGACTGAAGACGTACTACAA	15378–15401
NDiV-SZ-17-R	AGGCGTCAACAAGTGGTTGG	16499–16518
NDiV-SZ-18-F	CTGCCTAATGCTAACTGCTCTACC	16305–16328
NDiV-SZ-18-R	CTAAGCTGTGATTGTAAGTCGTGGA	17095–17119
NDiV-SZ-19-F	CACTTGTATCTAGCGCCGCTAA	16975–16996
NDiV-SZ-19-R	TTGCGTCATTGACACGTAGG	18026–18045
NDiV-SZ-20-F	TGCAACTCTACAGTCCAGATCC	17928–17949
NDiV-SZ-20-R	GGAGGAGCGTATAACTGCCGT	18838–18858
NDiV-SZ-21-F	TGGCGGCTAGCGTCACACTT	18688–18707
NDiV-SZ-21-R	AAGAATGGCCGGTACAATGTG	19642–19662
NDiV-SZ-22-F	GCCTGGAACAAGCCGATCA	19521–19539
NDiV-SZ-22-R	TTTACCTGTAATGCCAAGCGCC	20106–20127

Figure S1

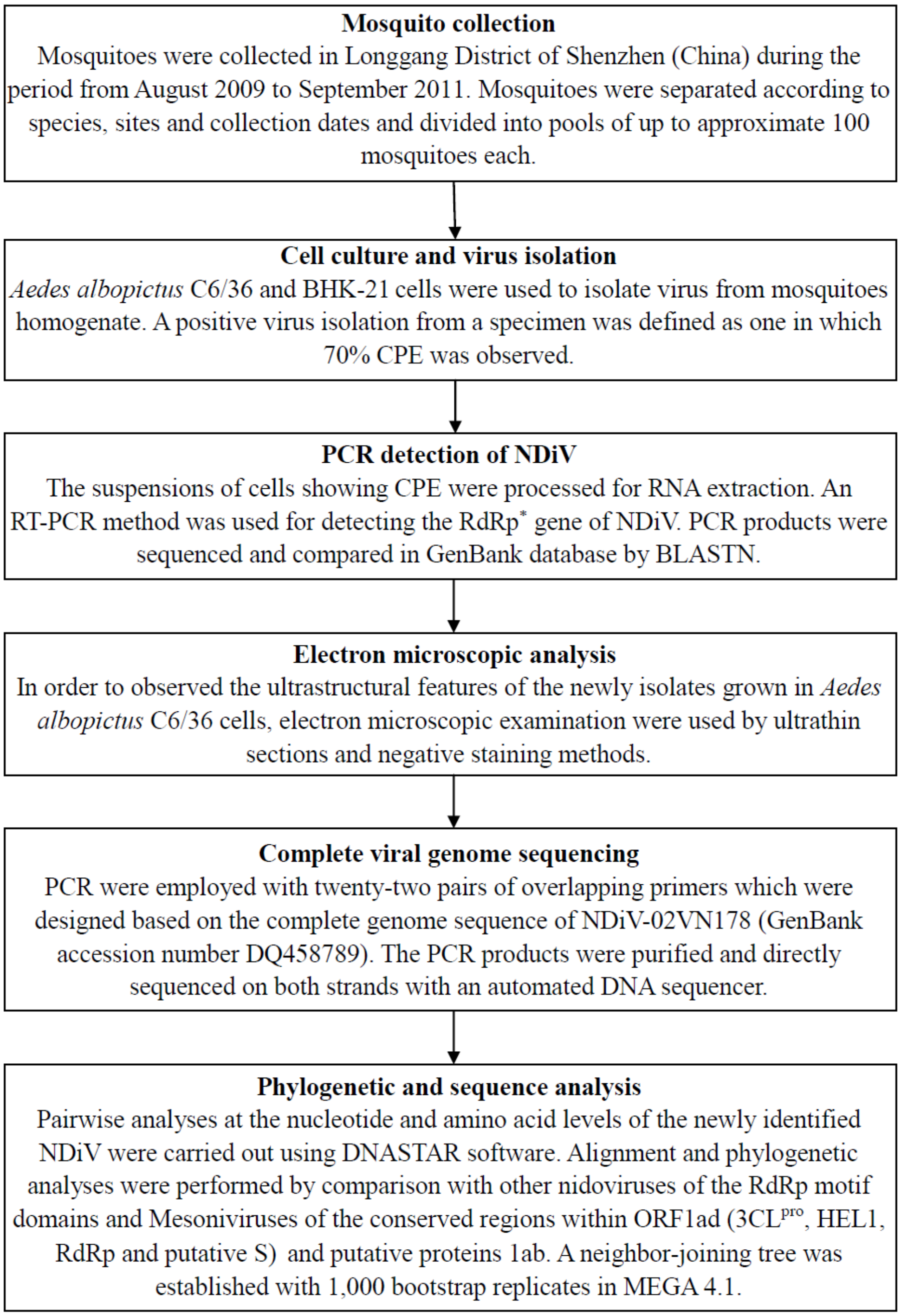

Figure S1. The flowchart for isolation and identification of Nam Dinh virus. * BHK-21, baby hamster kidney; CPE, Cytopathic effects; RT-PCR, reverse transcription-polymerase chain reaction; RdRp, RNA-dependent RNA-polymerase; NDiV, Nam Dinh virus; PCR, polymerase chain reaction; $3CL^{pro}$, 3C-like chymotrypsin-like protease; HEL1, Superfamily 1 Helicase; S, spike glycoprotein.
